# Pseudoexfoliation syndrome in a 27-year-old patient: a case report

**DOI:** 10.5935/0004-2749.20210041

**Published:** 2021

**Authors:** Agustín Nicolás Lucas, Irene Copati, Delia Sivori, Amund Ringvold

**Affiliations:** 1 Hospital Oftalmológico Dr. Pedro Lagleyze, Buenos Aires, Argentina; 2 Eye Department, University of Oslo, Oslo, Norway

**Keywords:** Exfoliation syndrome, Glaucoma, Cataract extraction, Young adult, Síndrome de exfoliação, Glaucoma, Extração de catarata, Adulto jovem

## Abstract

Pseudoexfoliation syndrome is more frequent in people aged >50 yeears, and its prevalence increases with age. Few reports have described cases in younger patients, all with a history of ocular surgery, especially iris resection. Herein, we describe the case of a 27-year old man with bilateral advanced glaucoma and pseudoexfoliation material in OS. He had undergone cataract surgeries OU and a penetrating keratoplasty OD during childhood. Currently, he presented with an intraocular pressure of 40 mmHg OU. The OS showed a white flaky material in the pupillary rim and anterior capsule and a Sampaolesi line as a gonioscopic finding. Trabeculectomy was performed OU, and intraocular pressure control was achieved. Unlike other previously reported cases, this patient did not present any apparent iris manipulation in the affected eye. However, he did undergo an iridectomy in the contralateral eye. This is also the first case to be accompanied by bilateral glaucoma at the time of detection of the pseudoexfoliation material.

## Abstract (pt)

A síndrome de pseudoesfoliação é mais frequente em pessoas com mais de 50 anos e sua prevalência aumenta com a idade. Poucos relatos descrevem casos em pacientes mais jovens, todos com história de cirurgia ocular, especialmente ressecção da íris. Descrevemos o caso de um homem de 27 anos com glaucoma bilateral avançado e material de pseudoesfoliação no OE. O paciente foi submetido a cirurgias de catarata em AO e a uma ceratoplastia penetrante no OD durante a infância. Atualmente, ele apresentou PIOs de 40 mmHg em AO. O OE apresentou material escamoso branco na borda pupilar e cápsula anterior, e linha Sampaolesi como achado gonioscópico. A trabeculectomia foi realizada em AO e obteve-se o controle da pressão intraocular. Diferentemente de outros casos relatados, o paciente não apresentou qualquer manipulação aparente da íris no olho afetado. No entanto, ele foi submetido a uma iridectomia no olho contralateral. Além disso, este é o primeiro caso a ser acompanhado de glaucoma bilateral no momento da detecção do material de pseudoesfoliação.

## INTRODUCTION

Pseudoexfoliation syndrome (XFS) is an age-related systemic microfibrillopathy with progressive accumulation of extracellular material in several tissues^(1)^. Ocular manifestations are generally bilateral but can be asymmetric, with the second eye being affected even several years later. It may be associated with glaucoma, with a risk of conversion of approximately 30% in a 10-year period, primarily occurring in the first 5 years^(1)^. It is more frequent in people aged >50 years, and age has been a consistent risk factor associated with XFS prevalence in most of the population studies^(2)^.

A few cases of XFS in patients aged <50 years have been reported in the literature, with all of them presenting with a history of intraocular surgery as a common factor^(3-7)^.

Herein, we report the case of a 27-year-old male patient with a history of cataract surgery in his childhood who currently presented with bilateral advanced glaucoma and the presence of unilateral pseudoexfoliation material (XFM) in the left eye.

## CASE REPORT

### Previous history

A 27-year-old man was previously treated at a different center from the age of 11 to 22 years. We collected the information available in the medical records. At age 11 years, he was diagnosed with type I diabetes. In the same year, cataract surgeries were performed on both eyes. It was not clear from the records whether the cataracts were congenital or secondary to diabetes. In the right eye (OD), phacoemulsification was performed with the placement of a 13-mm PMMA single-piece intraocular lens (IOL), and this eye presented a partial detachment of Descemet’s membrane and rupture of the posterior capsule as intrasurgical complications. In the left eye (OS), uncomplicated phacoemulsification was performed with the placement of a 13-mm PMMA single-piece IOL in the sulcus. Due to the detachment of Descemet’s membrane and subsequent corneal decompensation at age 13 years, a penetrating keratoplasty with an 8-mm button was performed in the OD. During these controls, the patient had no record of elevated intraocular pressure (IOP) values, use of antiglaucomatous treatment, or signs of glaucomatous damage or diabetic retinopathy. From age 22 to 27 years, he did not attend any ophthalmology practice.

### Present status

**Figure 1:**
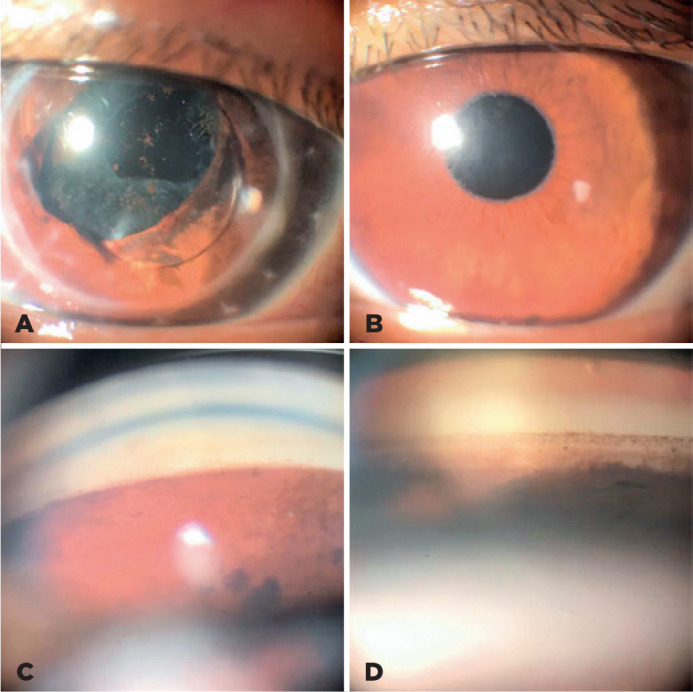
Comparison between the right (OD) and left (OS) eye. A) OD, transparent corneal button and pupillary capture of the IOL optic. B) OS, presence of white flaky material at the pupillary rim. C) OD gonioscopy, open angle with low pigmentation. D) OI gonioscopy, open angle with greater pigmentation and presence of Sampaolesi line.

**Figure 2:**
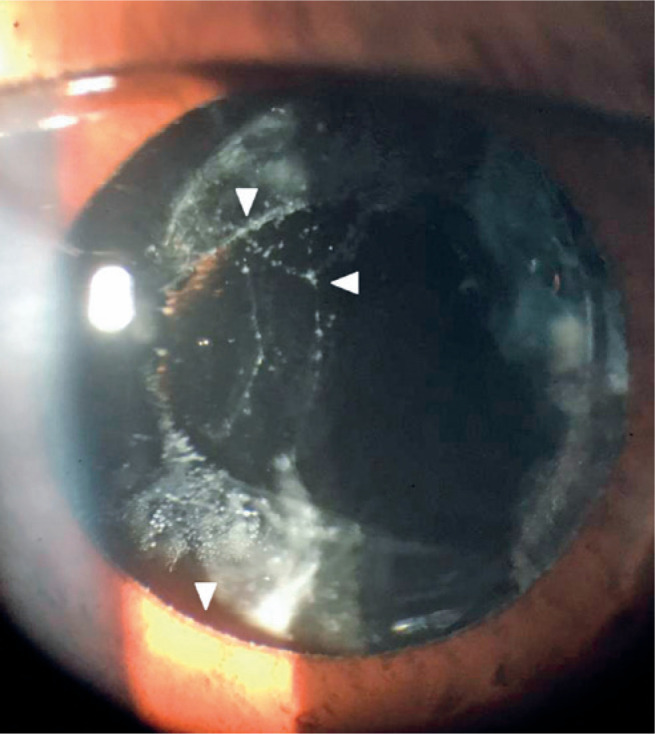
Slit-lamp examination of the left eye with pupil dilatation. A white flaky material is deposited at the pupillary rim, anterior capsule, and anterior vitreous face (arrows).

At the time of the visit to our center, the patient was aged 27 years. His ophthalmologic examination revealed a visual acuity of 20/1000 in the OD and 20/200 in the OS. The intraocular pressure was 40 mmHg in both eyes. In the slit-lamp evaluation, the OD presented a transparent corneal button and pupillary capture of the IOL optic. The OS presented a well-located IOL in the sulcus, and the presence of white flaky material in the pupillary rim, compatible with XFM (Figure 1). After pupillary dilatation, the same material was observed in the anterior capsule and anterior vitreous face (Figure 2); however, it was not detected in the OD. No rubeosis was observed in either eye. Gonioscopy revealed an open angle in both eyes, without the presence of neo-vessels. An asymmetry in pigmentation was noted, with greater pigmentation and presence of a Sampaolesi line in the OS. In the fundus examination, pale optic disks with 0.9 excavation and severe nonproliferative diabetic retinopathy were observed in both eyes. The pachymetry finding was 540 microns in both eyes.

The patient was treated with latanoprost and dorzolamide-timolol in both eyes, together with acetazolamide (250 mg every 12 h) and potassium supplement. Moreover, pan-photocoagulation with argon laser was performed in both eyes. However, only a suboptimal IOP reduction was achieved (OD 25 mmHg, OS 28 mmHg), and therefore, a trabeculectomy with mitomycin C as an adjuvant was performed on both eyes, starting with OS and with a 1-month interval between surgeries. Simultaneously, a conjunctival biopsy specimen was collected (2 × 2 mm) from the inferior nasal area in both eyes. No complications occurred during the interventions, and the patient evolved favorably. During the following 6 months of controls, the patient displayed unchanged visual acuity and an IOP of 12 mmHg in both eyes without topical treatment.

The conjunctival biopsy specimens were fixed with 2.5% glutaraldehyde, stained by uranyl acetate and lead citrate, and prepared for observation by transmission electron microscopy. Conventional connective tissue elements such as collagen, elastin, and fibroblasts were observed, but no XFM characteristic signs (such as aggregates of fibers in between normal connective tissue components or forming a ring-structure outside the vessel’s endothelium) were found in either eye.

## DISCUSSION

We describe a case of XFS in a 27-year old male patient. The diagnosis was based on slit-lamp examination of the left eye, which revealed white flaky material along the pupillary rim, anterior capsule, and anterior vitreous face. The diagnosis was further supported by the gonioscopic finding of a Sampaolesi line in the same eye.

The presence of XFM in the conjunctiva of eyes with XFS is well known^(8)^. However, it has also been demonstrated that XFM is unevenly distributed in the conjunctiva^(9)^. Therefore, it is possible to evaluate multiple blocks of tissue without detecting this typical material. The negative results observed in the present case do not rule out the diagnosis of XFS.

XFS is considered to be age-dependent. In the majority of epidemiological studies, all patients were aged >50 years^(2)^. As an exception, two studies have described cases in the age groups of 30-39 and 40-49 years. One study was conducted in the Pondo tribe of South African Bantu, and the other study included an Australian Aboriginal cohort^(2)^. The prevalence rates found among people aged <40 years were 1.3% in the Pondo tribe and 0.4% in the Australian Aboriginals. A limitation of both studies was the lack of strict criteria for the definition of XFS. In addition, no follow-up was conducted in either of these populations to evaluate the development of glaucoma.

Regarding the well-reported cases of XFS in patients aged <50 years, we found 9 case reports describing a total of 15 patients in the literature^(3-7)^. In 14 of these patients, the XFM was observed unilaterally, and only 1 patient had bilateral presence of XFM. All cases coincided in presenting a surgical history in the affected eye. In 14 of the 15 patients, the surgeries involved ma nipulation of the iris either by resection after a penetrating wound or by iridectomies in filtering surgeries or keratoplasties. It has been postulated that iris manipulation may be the trigger for early development in these cases^(5)^. An exception was a case with a history of uncomplicated extracapsular cataract surgery with IOL placement in the sulcus, where iris damage was not mentioned^(6)^. It has also been postulated that multiple intraocular surgeries can generate this situa tion^(7)^, although only 5 of 15 cases presented more than one surgery.

Our case matches with previous cases in terms of presenting a surgical history. With an uncomplicated phacoemulsification surgery in the affected eye, our case is the second to be described without apparent iris injury. However, the unaffected eye had a history of multiple surgeries, with an iridectomy performed alon gside a keratoplasty. Considering that XFS is a systemic disease, with bilateral and asymmetric compromise, it is possible to consider that manipulation of the iris of the contralateral eye had triggered the production of the material in the affected eye. No other report has suggested a similar process.

In 8 of the previous cases, there was a history of glaucoma before the detection of XFM. Among the remaining 7 cases without a previous glaucoma diagnosis, 3 cases developed unilateral glaucoma sometime after the detection of the material. Our patient had no signs of glaucoma before surgery. He presented with bilateral glaucoma and XFM in one eye by the time of our examination. We cannot assure that an association exists between the development of glaucoma and the presence of XFM in this case. Pseudophakic glaucoma caused by congenital cataract surgery is also a possibility in this patient.

Several genetic loci (LOXL1, CACNA1A, FLT1-POMP, TMEM136-ARHGEF12, AGPAT1, RBMS3, and SEMA6A) have been associated with an increased risk for XFS in elderly people^(10)^. Unfortunately, genetic studies were not available in our case.

In conclusion, XFS in patients aged <50 years is a rare occurrence, and the underlying physiopathology remains poorly understood. The most common hypothesis is that an iris resection might trigger the production of XFM. Regarding our case, although there was a history of intraocular surgery, iris manipulation did not occur in the affected eye.

As the contralateral eye had an iris resection, there is a possibility that this event could have triggered the production of material in the affected eye. However, we did not find sufficiently strong evidence to support this hypothesis.

It would be interesting to analyze whether some of these rare cases of XFS in young patients present any of the genetic variations described in elderly people.
